# Management of sexual dysfunction in breast cancer survivors: a systematic review

**DOI:** 10.1186/s40695-015-0009-4

**Published:** 2015-11-02

**Authors:** Susan M. Seav, Sally A. Dominick, Boris Stepanyuk, Jessica R. Gorman, Diana T. Chingos, Jennifer L. Ehren, Michael L. Krychman, H. Irene Su

**Affiliations:** 1 Department of Reproductive Medicine and Moores Cancer Center, University of California, San Diego, 3855 Health Sciences Dive #0901, La Jolla, CA 92093 USA; 2 Young Survival Coalition, 80 Broad Street, New York, NY 10004 USA; 3 University of California, Irvine, Beckman Laser Institute, 1002 Health Sciences Road, Irvine, CA 92612 USA; 4 Southern California Center for Sexual Health and Survivorship Medicine, 1501 Superior Avenue, Newport Beach, CA 92663 USA

**Keywords:** Breast cancer, Female sexual dysfunction, Systematic review, Cancer survivorship, Vaginal interventions, Sexual health

## Abstract

Female sexual dysfunction occurs frequently in midlife breast cancer survivors (BCS) and encompasses problems with sexual desire, interest, arousal, orgasm and genitopelvic pain. Although common, sexual problems are under-diagnosed and under-treated in BCS. The objective of this review was to assess primary studies that intervene on sexual dysfunction in BCS. In February 2015, PubMed, SCOPUS, CINAHL, COCHRANE and Web of Science databases were systematically searched for randomized controlled clinical trials (RCTs) of vaginal (lubricants, moisturizers, estrogens, dehydroepiandrosterone [DHEA], testosterone, vibrators, dilators), systemic (androgens, anti-depressants, flibanserin, ospemifene), physical therapy (physical activity, pelvic floor training), counseling and educational interventions on sexual function in BCS. Observational studies of vaginal interventions were also included due to the paucity of RCTs. The search yielded 1414 studies, 34 of which met inclusion criteria. Both interventions and outcomes, measured by 31 different sexual function scales, were heterogeneous, and therefore data were not pooled. The review found that regular and prolonged use of vaginal moisturizers was effective in improving vaginal dryness, dyspareunia, and sexual satisfaction. Educational and counseling interventions targeting sexual dysfunction showed consistent improvement in various aspects of sexual health. No consistent improvements in sexual health were observed with physical activity, transdermal testosterone or hot flash interventions. There was a lack of BCS-specific data on vaginal lubricants, vibrators, dilators, pelvic floor therapy, flibanserin or ospemifene. Overall, the quality of evidence for these studies was moderate to very low. Because each of the interventions with BCS data had limited efficacy, clinical trials to test novel interventions are needed to provide evidence-based clinical recommendations and improve sexual function in BCS.

## Introduction

In the United States, there are more than 2.3 million female cancer survivors who are younger than age 60; 40 % of these women are survivors of breast cancer [1]. Most midlife breast cancer survivors (BCS) undergo surgery, chemotherapy, radiation and/or endocrine therapy for cancer treatment. Receiving a breast cancer diagnosis and undergoing associated treatments including long term endocrine therapy can impair sexual function via a number of mechanisms, including disrupting ovarian function, body image, intimacy and relationships [2–7]. In turn, impaired sexual function contributes to lower quality of life in survivorship [8, 9].

Female sexual dysfunction has been classified into three categories: sexual interest or arousal disorder, orgasmic disorder, and genitopelvic pain or penetration disorder. A women is diagnosed with sexual dysfunction if she experiences persistent symptoms that last at least six months and cause marked distress, as detailed in the Diagnostic and Statistical Manual 5^th^ Edition (DSM-5) [10] (Table 1). A population-based cohort study of recently diagnosed BCS showed 65 % reported that they were sexually active; 52 % of sexually active women described problems with two or more areas of sexual function [11]. At 5 and 10 years after cancer diagnosis, prevalence of sexual problems remained significant, 26 and 19 %, respectively [12]. These findings that BCS are sexually active and experience sexual dysfunction that persists throughout survivorship have been replicated in multiple cohorts [9, 13–15].

**Table 1:** Female sexual dysfunction classification and diagnostic criteria from the Diagnostic and Statistical Manual of Mental Disorders, Fifth Edition [10]

Disorder^a^	Criteria
Female sexual interest or arousal disorder	Absent or significantly decreased sexual interest or arousal as manifested by a lack of or reduction in:
	1. Sexual activity
	2. Sexual or erotic thoughts or fantasies
	3. Initiation of sexual activity and unreceptive to partner’s attempts to initiate
	4. Sexual excitement or pleasure during sexual activity in at least 75 % of all sexual encounters
	5. Sexual interest or arousal in response to any internal or external sexual or erotic cues (written, verbal, or visual)
	6. Genital or non-genital sensations during sexual activity in at least 75 % of all sexual encounters
Female orgasmic disorder	Presence of either of the following in at least 75 % of all sexual activities:
	1. Significant delay in, frequency of, or absence of orgasm
	2. Significantly reduced intensity of orgasmic sensations
Genitopelvic pain or penetration disorder	Persistent or recurrent difficulties with one or more of the following:
	1. Vaginal penetration during intercourse
	2. Significant vulvovaginal or pelvic pain during intercourse or penetration attempts
	3. Significant fear or anxiety about vulvovaginal or pelvic pain in anticipation of, during, or because of vaginal penetration
	4. Significant tensing or tightening of pelvic floor muscles during attempted vaginal penetration

^a^Symptoms must persist for at least 6 months, cannot be attributed to another nonsexual mental disorder, are not related to or a result of relationship distress or other significant life stressors, and are not a consequence of the effects of a substance, medication, or other medical conditions

Sexual health is often under-addressed in survivorship care, and only a minority of BCS receives information and education about sexual function from oncology professionals [16]. Among primary care providers at a university-based medical center, 62 % self-reported never or rarely discussing sexual issues with cancer survivors [17]. Providers who perceived having adequate preparedness to evaluate late effects or formal training in survivorship care were more likely to address sexual health considerations. Conversely, lack of knowledge in healthcare providers was a significant barrier to discussions on sex [18]. Moreover, patients may be reluctant or embarrassed to raise sexual concerns with healthcare providers [19]. Only 50 % of BCS thought their providers were knowledgeable about cancer care follow-up and even fewer (41 %) felt that their providers were equipped to treat their cancer therapy-related symptoms [20]. Hence, disseminating evidence-based information on managing sexual concerns to healthcare providers is a critical aspect of improving sexual health care after breast cancer.

Multiple pharmacologic and behavioral treatments have been tested to improve sexual health after breast cancer. We present a systematic review of primary research on managing sexual dysfunction in breast cancer survivors to generate evidence-based content for improving knowledge on sexual health for BCS and their healthcare providers.

## Methods

### Search strategy

This systematic review was conducted in accordance with PRISMA guidelines [21]. In February 2015, we systematically searched the following databases: PubMed (1966 – February 2015), SCOPUS (1966 – February 2015), CINAHL (Cumulative Index to Nursing and Allied Health Literature) (1981 – February 2015), COCHRANE (all years), and Web of Science (1900 – February 2015). We screened the bibliographies of all included studies for additional references. We sought peer-reviewed articles examining interventions on sexual health among female BCS. We included studies on female breast cancer patients without age restriction and excluded studies on males, non-humans and other female cancer patients. We included studies on sexual dysfunction, including problems with dyspareunia, sexual pain, vaginismus, vaginal dryness, sexual arousal, desire, and orgasm. For types of interventions, we included vaginal (lubricants, moisturizers, estrogens, dehydroepiandrosterone [DHEA], testosterone, vibrators and dilators), systemic (androgens, anti-depressants, flibanserin, ospemifene), physical therapy (physical activity, pelvic floor training), counseling and educational interventions. We did not include studies on systemic estrogen interventions. For physical therapy, systemic, and counseling and educational interventions, we included only randomized controlled clinical trials (RCTs). We retained RCTs and observational studies (cohort and case control studies) on vaginal interventions due to the dearth of RCTs. We excluded qualitative studies and case reports. The final PubMed search strategy is detailed in the Appendix.

### Outcome measures

The primary outcome of this systematic review was sexual function. Measures of sexual function varied widely among studies and are summarized in Table 2.

**Table 2:** Sexual function outcome measures

	Assessment description^a^	Scoring rubric
Arizona Sexual Experience Scale (ASEX) [68, 69]	5-item scale measuring sexual drive, arousal, vaginal lubrication, orgasm, and satisfaction	• 6-point Likert scale (1 – 6); total score 5 – 30
		• Higher scores indicate greater sexual dysfunction.
		• Score > 19 indicates sexual dysfunction.
Body Image Relationships Scale (BIRS) [70]	11-item Appearance and Sexuality Subscale measuring satisfaction with sexual activity, physical appearance, and body image	• 5-point scale (1 – 5); total score 11 – 55
		• Higher scores indicate greater impairment.
Cancer Rehabilitation Evaluation System (CARES) [71]	Includes 4-item Sexual Interest, 4-item Sexual Function, and 18-item Marital Issues Subscales	• 5-point scale (0 – 4)
		• Higher scores indicate greater impairment.
Changes of Sexual Functioning Questionnaire (CFSQ) [72]	Includes 3-item Desire/Interest, and 2-item Frequency/Pleasure Subscales	• 5-point Likert scale (1 – 5); total score 5 – 25
		• Higher scores indicate lesser impairment.
Derogatis Inventory of Sexual Functioning (DISF-SR) [73]	4-item subscale measuring sexual drive and relationship satisfaction	• 5- or 9-point scale (depending on item)
		• Higher scores indicate less impairment.
EORTC Quality of Life Questionnaire (QLQ) Breast Cancer Module [74]	23-item Sexual Function and Body Image Subscales measuring breast cancer therapy side effects	• 4-point rating scale; total score 0 – 100 (after linear transformation)
		• Higher scores indicate less impairment.
Female Sexual Function Index (FSFI) [75]	19-item scale measuring sexual desire, arousal, lubrication, orgasm, satisfaction, and pain	• 6-point Likert scale (0 – 5); total score 0 – 36
		• Higher scores indicate better sexual function.
		• Score < 26.5 suggests sexual dysfunction.
Functional Assessment of Cancer Therapy (FACT) [76]	18-item Endocrine Symptoms Subscale (FACT-ES) measuring hormone-related/ menopausal symptoms of breast cancer	• Total score 0 – 72
		• Higher scores indicate fewer symptoms.
Marital Intimacy Questionnaire [77]	8-item scale measuring marital intimacy	• 4-point Likert scale (1 – 4); total score 8 – 32
		• Higher scores indicate less impairment.
Medical Outcomes Study [78]	4-item Sexual Functioning Subscale measuring sexual dysfunction symptoms	• 6-point Likert scale (1 – 6) • Higher scores indicate more symptoms.
Menopausal Sexual Interest Questionnaire (MSIQ) [79]	10-item Sexual Satisfaction Subscale measuring desire, responsiveness, and satisfaction in postmenopausal women	• 7-point Likert scale (1-7); total score 10 - 70 • Higher scores indicate less impairment.
Menopausal Symptom Score [80]	Adapted 7-item scale measuring study-specific menopausal symptoms	• 5-point Likert scale (0 – 4); total score 0 – 28
		• Higher scores indicate more symptoms.
Profile of Female Sexual Function (PFSF) [81]	37-item scale measuring sexual desire, arousal, orgasm, pleasure, concerns, responsiveness, and self-image	• 5-point Likert scale (1 – 5); total score 0 – 100 (after linear transformation)
		• Higher scores indicate less impairment.
Psychological Adjustment to Illness Scale (PAIS) [82]	46-item clinical interview with Sexual Relationships (PAIS-SR) and Sexual Problems Subscales measuring psychological and social adjustment to illness	• 4-point scale (0 – 3)
		• Higher scores indicate poorer adjustment.
		• Score < 35 = good; 35-51 = fair; > 51 = poor
Quality of Marriage Index (QMI) [83]	6-item scale measuring marital quality	• 7-point bipolar scale (1 – 7); total score 6 – 42
		• Higher scores indicate better quality of marriage.
Study-Specific Scales [50]	Study-specific scales measuring frequency of sexual desire, intercourse, masturbation, orgasm, initiative for sex, and relationship satisfaction	• Higher scores indicate less impairment.
Study-Specific Scales [51]	Study-specific items measuring sexual satisfaction, relationship satisfaction, dyspareunia, and comfort with sexuality	• 5- or 6-point Likert scale (depending on item)
		• Higher scores indicate less impairment.
Sexual Activity Questionnaire (SAQ) [84]	10-item scale with 3 main subscales measuring: Pleasure (SAQ-P; desire, enjoyment, satisfaction), Discomfort (SAQ-D; vaginal dryness, dyspareunia), Habit Subscale (SAQ-H; frequency)	• 4-point Likert scale (0 – 3); total score 0 – 24
		• Higher scores indicate less impairment.
Sexual Desire Subscale of Brief Index of Sexual Function (BISF) [85]	Includes 8-item Sexual Desire and 9-item Sexual Arousal Subscales	• 6- or 7-point Likert scale (0 – 5 or 0 – 6)
		• Higher scores indicate less impairment.
Sexual Dysfunction Scale [57]	25-item study-specific scale with 3 subscales measuring: Behavioral (vaginal dryness, dyspareunia, and frequency), Evaluative (interest, arousal, and satisfaction), Body image (sense of attractiveness, impact of weight change and hair loss)	• Total score 0 – 100
		• Higher scores indicate less impairment.
Sexual Function Subscale of Greene Climacteric Scale (GCS) [86]	21-item scale with 4 subscales measuring: Vasomotor Symptoms (2 items), Somatic Symptoms (7 items; headaches and muscle/joint pains), Psychological Symptoms (11 items), Sexual Function (1 item; sexual interest)	• 4-point scale (0 – 3); total score 0 – 6 (vasomotor), 0 – 21 (somatic), 0 – 33 (psychological), 0 – 3 sexual function; combined total score 0 – 63
		• Higher scores indicate more symptoms.
Sexual Problems Frequency [45]	Adapted subscale from BIRS measuring frequency of sexual problems	• 5-point Likert scale (0 – 4)
		• Higher scores indicate greater impairment.
Sexual Satisfaction Scale (SSS) [61, 87]	Study-specific items measuring relational sexual satisfaction for male and female partners	• Higher scores indicate less impairment.
Sexual Satisfaction Scale (SSS) [88]	17-item study-specific scale measuring sexual satisfaction among Korean women	• 4-point Likert scale (1 – 4); total score 17 – 68
		• Higher scores indicate less impairment.
Sexual Self Schema Scale [89]	50- item, trait-adjectives scale measuring women’s sexual self-perception	• 7-point Likert scale (0 – 6)
		• Higher scores indicate better self-perception.
Visual Analog Scale for Vaginal Dryness and Dyspareunia [29]	Linear rating scale measuring vaginal dryness and pain	• 10-point scale (0 – 10)
		• High scores indicate greater impairment.
Vaginal Atrophy Symptom [29, 90–92]	Adapted 3-item scale measuring vaginal dryness, itching/irritation, dyspareunia	• 4-point Likert scale (0 – 3); total score 0 – 9
		• Higher scores indicate greater impairment.
Vaginal Dryness, Vaginal Itching, Dyspareunia [23, 32]	Study-specific items measuring vaginal dryness, vaginal itching, and dyspareunia	• 5- or 10-point Likert scale (depending on study)
		• Higher scores indicate greater impairment.
Vaginal Health Index (VHI) [93, 94]	6-parameter gynecological examination rating appearance of vaginal mucosa	• Score of 1 to 5; total score 6 – 30
		• Higher index indicates healthier appearance.
Vaginal Maturation Index (VMI) [91, 95]	Gynecological examination determining vaginal atrophy	• Score of 0 – 100 (%)
		• Higher scores indicate less vaginal atrophy.
		• A score of < 50 indicates vaginal atrophy.
Vaginal Symptoms Score (VSS) [24, 95]	Study-specific scale measuring severity of vaginal atrophy	• 5-point Likert scale (0 – 4)
		• Higher scores indicate more symptoms.

^a^All scales were designed as self-report questionnaires (unless otherwise reported as an examination, interview, or visual analog scale)

### Data collection

Three review authors (SS, SD, IS) independently screened the titles and abstracts of all search citations using the inclusion and exclusion criteria. Discrepancies among authors were resolved via consensus. Two of the three review authors (SS, SD, or IS) independently abstracted data on included articles. Data extracted included participants, interventions, sexual health outcome measures, results, and risks of bias (randomization, allocation concealment, blinding, sample size and analysis approach).

Risk of bias for all included studies was assessed independently by two review authors (SD and IS) using the Cochrane risk of bias assessment tool [22]. Discrepancies were resolved by discussion. Studies were evaluated for the following: selection bias (random sequence generation and allocation concealment); performance blinding (blinding of participants and personnel); detection bias (blinding of outcome assessment); attrition bias (incomplete outcome data); reporting bias (selective reporting); and other bias. Each bias criteria was assigned a high, low or unclear risk of bias rating. Additionally, we evaluated the quality of each study using the following GRADE criteria: study limitations (i.e., risk of bias); consistency of effect; imprecision; indirectness and publication bias. RCTs were first classified as high quality, and observational studies were first classified as low quality. All studies were downgraded in quality for any of the following problems: serious limitation to study quality; important inconsistency; uncertainty about directness; imprecise or sparse data; or high probability of reporting bias.

## Results

**Fig. 1:**
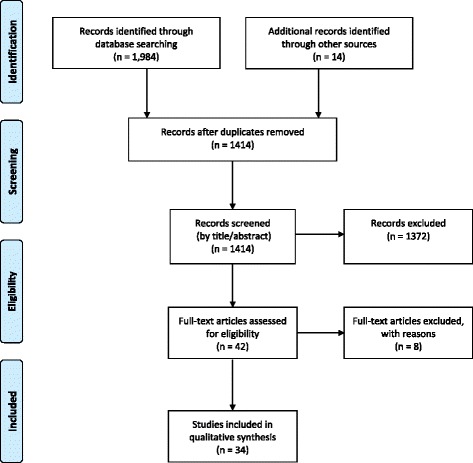
PRISMA Flow Diagram [21]. Description of articles included throughout the different phases of the systematic review

After searching PubMed (n = 637), SCOPUS (n = 665), CINAHL (n = 276), COCHRANE (n = 220) and Web of Science (n = 186) and hand picking (n = 14), 1984 articles were retrieved, leaving 1414 articles after removing duplicates. Forty-two full-text articles were accessed, from which 8 were excluded, leaving 34 articles included in this review. The PRISMA flow diagram details study selection results (Fig. 1). No article was excluded because of non-English language.

A total of 31 different sexual health outcome measures were used to assess intervention effects across the 34 papers (Table 2). The Female Sexual Function Index (n = 4 studies) and Cancer Rehabilitation Evaluation System (n = 3 studies) were the most commonly used measures. The Vaginal Maturation Index, Vaginal Health Index, and Sexual Activity Questionnaire were each used in 2 separate studies. All other outcome measures were used by single studies. Because of heterogeneity in both intervention and outcome measures, we were unable to pool estimates for a meta-analysis or derive strengths of recommendations based on the GRADE approach.

### Vaginal products interventions

**Fig. 2:**
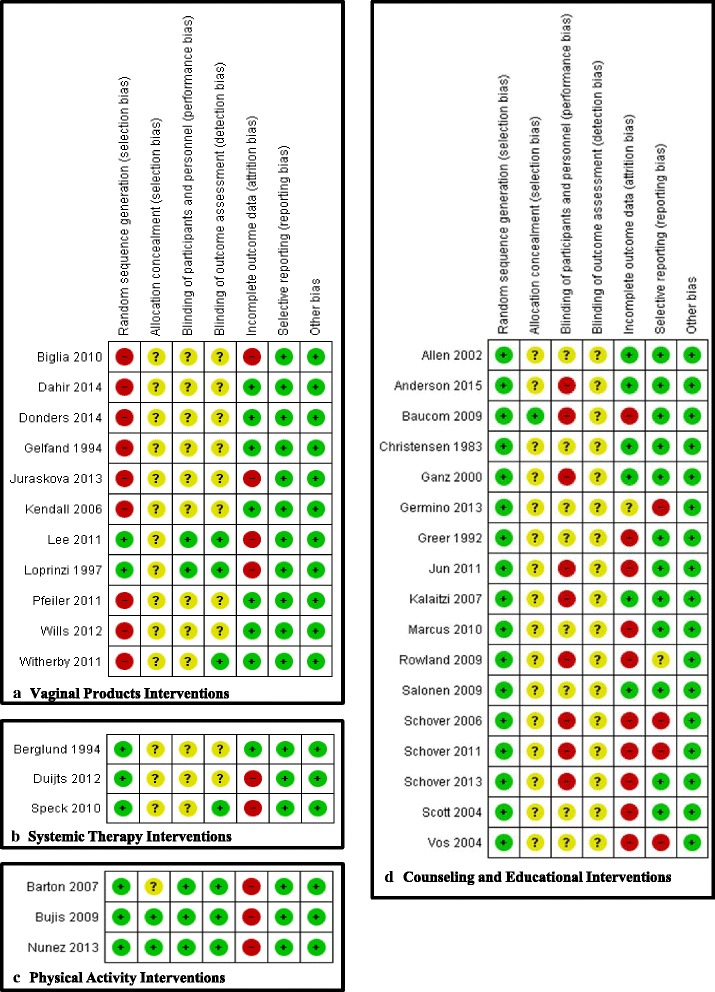
Risk of Bias Summary. Risk of bias figures detailing the review authors’ judgements about each risk of bias item for each included study organized by type of intervention: **a** Vaginal products interventions, **b** Systemic therapy interventions, **c** Physical activity interventions, **d** Counseling and educational interventions

We searched for studies on vaginal lubricants, moisturizers, estrogens, DHEA, testosterone, vibrators and dilators. Eleven studies met inclusion criteria (Tables 3 and 4, Fig 2a). No studies were found on lubricants, DHEA, vibrators and dilators. There were 3 RCTs and 8 single-arm prospective cohorts with no controls. All participants had genitourinary symptoms, experienced ≥ 6 months of amenorrhea, and completed primary breast cancer treatment. The studies occurred in Australia, Belgium, Germany, Italy, Korea, and the United States. The polycarbophil-based moisturizer Replens® was tested in 4 studies involving 133 participants, one in combination with olive oil and pelvic floor muscle relaxation [23–26]; compounded testosterone cream was tested in 2 studies involving 34 participants [27, 28]; pH balanced lactic acid gel was used in 1 study of 98 participants [29]; and vaginal estrogens were used in 5 studies involving 47 participants [24, 30–33]. Outcomes included patient-reported vaginal symptoms, such as dryness, dyspareunia and itching, and vaginal exam-based pH and cytology.

**Table 3:** Summary of studies

Vaginal products interventions					
Reference	Study design	Participants^a^	Intervention description	Intervention frequency	Outcome measures
Biglia (2010) [24]	Prospective cohort study	• Sample size = 31	Two groups:	12-week intervention:	1. Vaginal Symptoms Score
		• Mean age 54.1 in estrogen groups; 46.1 in polycarbophil-based moisturizer group	1. Estriol cream 0.25 mg (Angelini®) or micronized estradiol tablet 12.5 mcg (Vagifem®)	• Product twice weekly	2. Profile of Female Sexual Function
					3. Vaginal Health Index
			2. Polycarbophil-based moisturizer 2.5 g (Replens®)		
Dahir (2014) [27]	Prospective cohort study	• Sample size = 13	One group:	4-week intervention:	1. Sexual function (FSFI)
		• Mean age 59.7	1. Vaginal compounded testosterone 300 mcg in 0.5 mL	• Daily for 4 weeks	
		• Aromatase inhibitor treatment			
Donders (2014) [32]	Prospective cohort study	• Sample size = 16	One group:	12-week intervention:	1. Serum estradiol, estrone
		• Mean age 57.0, range 52-63	1. Tablet with 0.03 mg estriol and *L. acidophilus* (Gynoflor®)	• Daily for 4 weeks, then 3 times weekly for 8 weeks	
		• Aromatase inhibitor treatment			2. Serum estriol
					3. Vaginal symptoms
					4. Vaginal pH
					5. Vaginal maturation index
					6. Serum FSH
					7. Serum LH
Gelfand (1994) [25]	Prospective cohort study	• Sample size = 25	One group:	12-week intervention:	1. Vaginal health index
		• Mean age 60.1, range 43-78	1. Polycarbophil-based moisturizer 2.5 g (Replens®)	• Moisturizer three times weekly	2. Vaginal pH
					3. Patient-reported sexual effects
Juraskova (2013) [26]	Prospective cohort study	• Sample size = 25	One group:	26-week intervention:	1. Dyspareunia
		• Mean age = 51, range 37-66	1. Polycarbophil-based moisturizer 2.5 g (Replens®); Pelvic floor muscle relaxation; and Organic olive oil	• Moisturizer three times weekly	2. Sexual Activity Questionnaire
		• In a sexual relationship		• Pelvic floor muscle relaxation twice daily	3. Sexual satisfaction (FSFI subscale)
				• Olive oil use with intercourse	4. Endocrine symptoms (FACT-ES)
					5. Satisfaction and acceptability
Kendall (2006) [31]	Prospective cohort study	• Sample size = 6	One group:	12-week intervention:	1. Atrophic vaginitis symptoms
		• Mean age = 52, range 51-59	1. Micronized estradiol 25 mcg (Vagifem®)	• Daily for 2 weeks then twice weekly	
					2. Serum estradiol
		• Aromatase inhibitor treatment			3. Serum FSH
					4. Serum LH
Lee (2011) [29]	Randomized controlled trial	• Sample size = 98	Two groups:	12-week intervention:	1. Dryness with pain
		• Mean age 45.9, range 34-53 in intervention group; 45.0, range 37-53 in placebo group	1. pH-balanced lactic acid gel (pH 4.0)	• Gel three times weekly	
					2. Dyspareunia
	• Double blind		2. Placebo gel (pH 7.2)		3. Vaginal health index
	• Placebo control				4. Vaginal pH
		• Pre-menopause status prior to breast cancer diagnosis			5. Vaginal maturation index
Loprinzi (1997) [23]	Randomized controlled trial	• Sample size = 52	Two groups:	9-week intervention:	1. Product preference
		• Age ≤ 45 = 22 %	1. Polycarbophil-based moisturizer 2.5 g (Replens®), then	• First product: daily x 5 days, three times weekly x 23 days	2. Vaginal dryness
		Age 46-55 = 38 %			3. Dyspareunia
	• Double blind				
	• Cross-over	Age ≥ 56 = 40 %	Placebo (Hydroxymethylcellulose, glycerine-delta lactone, hydrogenated palm oil glyceride, water)	• 1 week washout	4. Itching
				• Second product: daily x 5 days, three times weekly x 23 days	
			2. Placebo, then Replens®		
Pfeiler (2011) [30]	Prospective cohort study	• Sample size = 10	One group:	2-week intervention:	1. Vaginal dryness
		• Mean age 65, range 50-77	1. Estriol 0.5 mg vaginal tablet	• Daily for 2 weeks	2. Dyspareunia
					3. Serum estradiol
		• Aromatase inhibitor treatment			4. Serum FSH
					5. Serum LH
Wills (2012) [33]	Cross-sectional study	• Sample size = 48	Three groups:	Ongoing interventions:	1. Serum estradiol
		• Mean age 60, range 49-67 in vaginal estrogen groups; 68, range 53-79 in control group	1. 25 mcg estradiol tablet (Vagifem®)	1. Twice weekly ongoing	
			2. Vaginal estrogen ring (Estring®)	2. Every 90 days ongoing	
			3. Control: no vaginal estrogen	3. No vaginal estrogen	
		• Aromatase inhibitor or SERM treatment			
Witherby (2011) [28]	Prospective cohort study	• Sample size = 21	Two groups:	4-week intervention:	1. Serum estradiol
		• Mean age 57, range 47-66 in 150 mcg group; 56, range 45-69 in 300 mcg group	1. Vaginal compounded testosterone 150 mcg in 1 g cream	• Daily for 4 weeks	2. Vaginal atrophy symptom
					3. Vaginal pH
					4. Vaginal maturation index
		• Aromatase inhibitor treatment	2. Vaginal compounded testosterone 300 mcg in 1 g cream		

^a^All studies required history of breast cancer; post menopause or ≥ 6 months of amenorrhea; genitourinary symptoms; and completion of primary cancer treatment for study participation

**Table 4:** Summary of findings

Vaginal products interventions						
Reference	Outcomes	Intervention results	Control results	Comparisons	Quality of evidence (GRADE)	Comments
Biglia (2010) [24]	1. Vaginal Symptoms Score	Baseline to 4-week score change (SD)	No control group	Between group comparisons	Very Low	• Dropout: 16 %
	2. Profile of Female Sexual Function (PFSF)					• Estrogens improved all outcomes more than Replens®.
				1. 4 weeks: *p* = 0.66		
		1. Replens®: - 6.3 (4.3)		12 weeks: *p* = 0.01		
	3. Vaginal Health Index (VHI)	Estrogens: -5.3 (4.7)		2. 12 weeks:		
		2. Not reported		*p* = 0.19		• Replens® showed no change in sexual function at 12 weeks.
		3. Replens®: +3.0 (1.6)		3. 4 weeks: *p* = 0.05		
		Estrogens: +5.9 (3.0)		12 weeks:		
		Baseline to 12-week score change (SD)				
				*p* = 0.02		• Serum estradiol increased 1.4-3.1 pg/mL in Estrogens group (*p* > 0.05).
		1. Replens®: -1.3 (5.5)		Within group comparisons versus baseline		
		Estrogens: -11.6 (5.2)				
		2. Replens®: +2.1 (9.3)		Replens®		
				1. 4 weeks: *p* = 0.01		
		Estrogens: +7.2 (5.4)				
		3. Replens®: +2.0 (3.4)		12 weeks: *p* = 0.72		
		Estrogens: +8.5 (3.6)		2. 12 weeks: *p* = 0.70		
				3. 4 weeks: *p* = 0.07		
				12 weeks: *p* = 0.42		
				Estrogens		
				1. 4 weeks: *p* < 0.01		
				12 weeks: *p* < 0.01		
				2. 12 weeks: *p* =0.03		
				3. 4 weeks: *p* < 0.01		
				12 weeks: *p* < 0.01		
Dahir (2014) [27]	1. Sexual function (FSFI)	Mean (SD)	No control	Within group comparisons versus baseline	Low	• Dropout: 8 %
		1. Pre 8.7 (3.8)				• Significant improvement in all FSFI domains by post-test.
		Post 18.8 (7.1)		1. *p* < 0.001		
Donders (2014) [32]	1. Serum estradiol, estrone	Baseline, 4-week	No control	Within group comparisons versus baseline	Low	• Dropout: None
	2. Serum estriol	1. Only 1 estradiol level detectable (1.2 pg/mL)				• 1 of 16 participants with detectable estradiol level (1.2 pg/mL) at day 28.
	3. Vaginal symptoms			1. Descriptive only		
		2. Peak estriol 104.5 pg/mL, 15.8 pg/mL		2. Descriptive only		
	4. Vaginal pH			3. *p* < 0.001 for dryness, soreness		
		3. Improved dryness, soreness, dyspareunia				
	5. Vaginal maturation index			4. *p* < 0.001		
	6. Serum FSH	4. Mean 6.0, 4.4		5. *p* < 0.001		
	7. Serum LH	5. 31 %, 72 %		6. *p* = 0.03		
		6. Mean 107.9, 98.9		7. *p* > 0.05		
		7. Mean 36.5, 34.0				
Gelfand (1994) [25]	1. Vaginal health index	1. Mean score (SD)	No control	Within group comparisons versus baseline	Low	• Dropout: None
	2. Vaginal pH	Baseline 10.1 (0.5)				• Vaginal irritation in 12 % of participants.
	3. Patient-reported sexual effects	1-month 10.8 (0.4)		1. 1-month		
		3-month 19.7 (0.7)		*p* > 0.05		
		2. Mean pH (SD)		>1 month		
		Baseline 6.9 (0.2)		*p* <0.001		
		1-month 6.8 (0.1)		2. 1-month		
		3-month 4.9 (0.2)		*p* >0.05		
		3. Pain-free intercourse:		>1 month		
		Baseline 36 %		*p* <0.001		
		4-month 69 %		3. Descriptive only		
		Sexual satisfaction improved:				
		1-month 0 %				
		3-month 77 %				
		Sexual frequency improved:				
		1-month 0 %				
		3-month 42 %				
Juraskova (2013) [26]	1. Dyspareunia (Visual analog score, 0-10)	Mean (SD)	No control	Within group comparisons versus baseline	Very Low	• Dropout: 36 %
		1. Baseline 7.0 (2.4)				• Maximum gain in sexual satisfaction and dyspareunia occurred by 12 weeks.
	2. Sexual Activity Questionnaire (0-24)	4-week 4.4 (2.4)		1. *p* < 0.001		
		26-week 2.7 (2.3)		2. *p* < 0.001		
	3. Sexual satisfaction FSFI subscale (0.8-6)	2. Baseline 7.2 (3.2)		3. *p* < 0.001		
		4-week 12.3 (4.3)		4. *p* = 0.01		
	4. Endocrine symptoms (FACT-ES, 0-72)	26-week 11.6 (4.3)		5. Descriptive only		
		3. Baseline 2.4 (1.4)				
	5. Satisfaction and acceptability	4-week 3.3 (1.8)				
		26-week (3.5 (1.4)				
		4. Baseline 51 (9.2)				
		4-week 51.8 (9.9)				
		26-week 53.8 (8.7)				
		5. Intervention helpful:				
		PFM 92 %				
		Replens® 88 %				
		Olive oil 76 %				
Kendall (2006) [31]	1. Atrophic vaginitis symptoms (yes/no)	1. 5 of 6 improved	No control	Descriptive data – no comparisons	Low	• Dropout: None
	2. Serum estradiol, pmol/L	2. 5 of 6 had estradiol levels > 3 pmol/L				
	3. Serum FSH, IU/l	3. No significant change				
	4. Serum LH, IU/l	4. No significant change				
Lee (2011) [29]	1. Dryness with pain (Visual analog score 0-10)	Baseline, 12-week mean scores (SD)	Baseline, 12-week mean scores (SD)	Between group comparisons	Moderate	• Dropout: 12 %
				1. *p* = 0.001		• All outcomes favor intervention.
	2. Dyspareunia (Visual analog score 0-10)	1. 8.2 (0.8),	1. 7.9 (0.9),	2. *p* = 0.04		
		4.2 (1.4)	6.5 (1.5)	3. *p* = 0.002		• Vaginal irritation in 50 % participants in first 4 weeks.
	3. Vaginal health index	2. 8.2 (1.0),	2. 8.1 (1.0),	4. *p* < 0.001		
	4. Vaginal pH	5.5 (1.1)	6.1 (1.4)	5. *p* < 0.001		
	5. Vaginal maturation index	3. 15.8 (3.7), 21.0 (3.9)	3. 14.3 (3.7), 17.0 (3.9)			
		4. 6.5 (1.1),	4. 6.2 (1.1),			
		5.0 (0.8)	5.7 (0.9)			
		5. 45.5 (3.5), 51.2 (3.8)	5. 46.4 (3.7), 47.9 (2.7)			
Loprinzi (1997) [23]	1. Product preference	1. 41 % prefer Replens® % score decrease after 4 weeks treatment:	1. 24 % prefer Placebo % score decrease after 4 weeks treatment:	Between group comparisons	Moderate	• Dropout: 27 %
	2. Vaginal dryness (scale 0-4)			1. *p* = 0.68		• Both groups with improved vaginal dryness by 1 week.
				2. *p* = 0.3		
	3. Dyspareunia (scale 0-4)	2. 64 %	2. 62 %	3. *p* = 0.05		
	4. Itching (scale 0-4)	3. 60 %	3. 41 %	4. Not reported		• Vaginal side effects in 42 % participants.
		4. Not reported	4. Not reported			
Pfeiler (2011) [30]	1. Vaginal dryness (yes/no)	1. 5/6 reported improvement	No control	Within group comparisons versus baseline	Low	• Dropout: None
	2. Dyspareunia (yes/no)	2. 3/5 reported improvement				
	3. Serum estradiol, pg/mL	3. All estradiol < 10 pg/mL after treatment		1. Descriptive only		
	4. Serum FSH, mU/mL			2. Descriptive only		
	5. Serum LH, mU/mL	Mean level pre-, post-		3. Descriptive only		
		4. 4. 75.7, 66.0		4. *p* = 0.01		
		5. 5. 32.4, 28.9		5. *p* = 0.02		
Wills (2012) [33]	1. Serum estradiol	1. Median level (95 % CI) pre-, post-	1. Mean (range) 3.72 pmol/L (3.0-7.7)	Between group comparisons pre-, post-	Low	• Dropout: None
						• Systemic absorption occurs with intravaginal estrogen therapy (ring or tablet).
		- Vaginal tablet 2.9 pmol/L (2.9-4.9), 45 pmol/L (19-89)		1. Versus vaginal tablet *p* = 0.93, *p* < 0.001;		
		- Vaginal ring 15.0 pmol/L (2.9-19), 15 pmol/L (1.9-35)		Versus vaginal ring *p* < 0.014, *p* < 0.014		
Witherby (2011) [28]	1. Serum estradiol	1. % < 5 pg/mL	No control	1. *p* = 0.91	Low	• Dropout: 10 %
	2. Vaginal atrophy symptom (Likert scale, 0-12)	Baseline 100 %		2. *p* < 0.001		• Two estradiol levels elevated after testosterone (both <8 pg/mL).
		4-week 90 %		3. *p* = 0.03		
	3. Vaginal pH	2. Mean (SD)		4. *p* < 0.001		
	4. Vaginal maturation index (VMI)	Baseline 5.9 (1.9)				• Adverse effects: hair growth/acne (n = 3), vaginal irritation (n = 3)
		4 week 2.1 (1.8)				
		3. Median				
		Baseline 5.5				
		4-week 5.0				
		4. % VMI ≥ 10				
		Baseline 20 %				
		4-week 40 %

In women using Replens®^,^ vaginal dryness decreased in the first week of use [23], with significant additional improvement in dryness, dyspareunia, sexual satisfaction and frequency by 4 and 12 weeks of use [23, 25, 26]. Compared with local vaginal estrogens (estriol or estradiol), Replens® appeared less effective at decreasing vaginal symptoms and improving vaginal histology. However, women who used vaginal estrogens experienced an increase in their serum estradiol levels or decline in gonadotropins, both evidence of systemic absorption [24, 30–33]. At steady state, women on aromatase inhibitors using 25 microgram estradiol tablets twice weekly had low levels of serum estradiol (median 1.3 pg/mL) [33]. However, 12 h after insertion of the tablet, median peak estradiol reached approximately 28 pg/mL [33]. A pH-balanced gel (pH 4.0) decreased vaginal dryness and dyspareunia more than the placebo gel with a higher pH [29]. Across products, vaginal irritation occurred in 12-50 % of participants, but whether this symptom persisted was not well described.

Two studies without control participants intervened with vaginal compounded testosterone in BCS on aromatase inhibitors [27, 28]. Compared to baseline measures, 4 weeks of vaginal testosterone improved all domains of the Female Sexual Function Inventory (FSFI) and vaginal atrophy symptoms. One study found 10 % (n = 2) of women had detectable serum estradiol levels after testosterone, though both estradiol levels were very low, <8 pg/mL [28].

### Systemic therapy interventions

We sought studies using systemic androgens, anti-depressants, ospemifene and flibanserin to intervene on sexual function (Tables 5 and 6, Fig 2b). No studies on ospemifene or flibanserin were found. Three randomized, double-blind cross-over trials on androgens and anti-depressants were included. All participants completed primary cancer treatment. The studies were conducted in Brazil, Netherlands, and the United States. In the single study on applying daily testosterone cream to the skin for one month, testosterone in postmenopausal cancer survivors did not result in greater sexual desire, pleasure or function than placebo cream [34]. This study accepted all cancer types, with 73 % of the 150 participants on tamoxifen or aromatase inhibitor, suggesting that they are breast cancer survivors. No increases in estradiol were noted while on testosterone cream, consistent with prior studies in women without history of breast cancer [35–40]. Two additional trials involving 115 participants intervened on hot flashes as the primary outcome with venlafaxine, clonidine or bupropion and examined if sexual function differed by these medications [41, 42]. There were no differences in sexual function between women treated with venlafaxine compared to clonidine nor with women treated with bupropion versus placebo [41, 42].

**Table 5:** Summary of studies

Systemic therapy interventions					
Reference	Objective	Participants^a^	Intervention description	Intervention frequency	Outcome measures
Barton (2007) [34]	To test transdermal testosterone for increasing sexual desire	• Sample size = 150	Two groups:	8-week intervention:	1. Sexual desire (CSFQ desire subscale)
		• Mean age 52.3 (SD 7.9)	1. Vanicream + 2 % testosterone (T), then Vanicream (placebo)	• First product: daily x 4 weeks	2. Pleasure (CSFQ pleasure subscale)
		• Post-menopause status		• Second product: daily x 4 weeks	3. Sexual function (CSFQ total score)
		• History of any cancer (73 % breast cancer)	2. Vanicream (placebo), then Vanicream + 2 % testosterone cream (T)		4. Serum testosterone
					5. Serum estradiol
		• Decreased sexual desire (Changes of Sexual Functioning Questionnaire, CSFQ)	• Testosterone dose 10.4 mg daily		6. Serum SHBG
					7. Serum AST
Buijs (2009) [42]	To test venlafaxine versus clonidine on hot flashes	• Sample size = 60	Two groups:	18-week intervention:	1. Sexual function (Sexual Activity Questionnaire)
		• Median age 49-51, range 35-60	1. Clonidine (C) then Venlafaxine (V)	• One medication daily x 8 weeks	
		• History of breast cancer	2. Venlafaxine, then Clonidine or vice versa	• 2-week washout	
		• ≥ 14 hot flashes/week		• Second medication daily x 8 weeks	
			• V: 75 mg once daily		
			• C: 0.05 mg twice daily		
Nunez (2013) [41]	To test bupropion on hot flashes	• Sample size = 55	Two groups:	10-week intervention:	1. Sexual function (Arizona Sexual Experience Scale)
		• Median age 49, range 33-71	1. Bupropion (B), then placebo	• One medication daily x 3 days, then twice daily for 25 days	
		• History of breast cancer	2. Placebo then bupropion		
		• ≥ 7 hot flashes/week	• B: titrated to 300 mg daily	• 1-week washout	
				• Second medication daily x 3 days, then twice daily for 25 days	
Physical Activity Interventions					
Berglund (1994) [45]	To test physical training, information and coping skills training on physical strength, information need and mood symptoms	• Sample size = 199	Two groups:	7-week intervention, 3-months follow up:	1. Sexual problems frequency
		• All cancers (80 % breast cancer)	1. Structured rehabilitation run by oncology nurse:	1. Twice-weekly 2-h sessions for first 4 weeks, then one-weekly 2-h session for 3 weeks	
		• Within 2 months of completing primary cancer treatment			
			Group sessions on physical training, cancer information, coping training		
				2. 0 or 1 information session	
			2. Controls: Cancer information session with oncologist/ dietitian		
Duijts (2012) [43]	To test cognitive behavioral therapy and physical exercise on menopausal symptoms	• Sample size = 422	Four groups:	12-week intervention:	1. Sexual function (Sexual Activity Questionnaire, habit subscale)
		• Mean age 48.2 (SD 5.6)	1. Cognitive behavioral therapy (CBT): Group sessions on reducing menopausal symptoms	1. 6 weekly groups and 12^th^ week refresher	
		• ≥ 2 menopausal symptoms over prior 2 weeks			
				2. Physiotherapist follow up in weeks 4 and 8	
			2. Physical exercise (PE): Home-based, self-directed exercise program to achieve target heart rates, tailored at start by physiotherapist.		
			3. CBT/PE		
			4. Waitlist control		
Speck (2010) [44]	To test strength training on perceptions of body image	• Sample size = 295	Two groups:	1-year intervention:	1. Self-perception of appearance and sexuality (Body Image Relationships Scale, appearance and sexuality subscale)
		• Mean Age 56.5 (SD 9)	1. Weight-lifting group instruction at community fitness center on warm-up, core exercises, weight-lifting exercises	1. First 13 weeks: twice weekly group sessions	
		• Lymphedema or at risk for lymphedema			
				Remaining of year: twice weekly unsupervised exercise; Fitness trainers called participants if they missed sessions.	
			2. Waitlist control		
				2. Maintain usual exercise level	
Individual-based Counseling and Educational Interventions					
Allen (2002) [55]	To test problem-solving therapy for problems and emotional difficulties	• Sample size = 164	Two groups:	16-week intervention:	1. Sexual health (CARES sexual subscale)
		• Mean age 42.3 (SD 5.4)	1. Problem-solving therapy: Training sessions and manual on problem solving targeting mid-life breast cancer patients	1. 6 biweekly training sessions with nurse: 2 in person, 4 on telephone	
		• Initiating chemotherapy			2. Marital relationship (CARES marital subscale)
			2. Controls: No therapy		
Anderson (2015) [46]	To test a behavioral intervention on menopausal symptoms	• Sample size = 55	Two groups:	12-week intervention:	1. Sexual function (sexual function subscale, Greene Climacteric Scale)
		• Mean age 49.2 (SD 6.2)	1. Multi-modal tailored program:	1. Nurse consultation at weeks 1, 6, 12	
		• < 12 months from primary cancer treatment	Goal setting in consultation with nurse, follow up calls and emails, written health education, newsletters and website		
		• ≥ 1 menopausal symptom as moderate or severe			
			2. Controls: Booklet on breast cancer and early menopause		
Ganz (2000) [48]	To test behavioral and non-estrogen replacement pharmaco-logic interventions on menopausal symptoms	• Sample size = 76	Two groups:	4-month intervention:	1. Menopausal Symptom Scale Score (hot flash, vaginal and urinary subscales of BCPT Symptom Checklist)
		• Mean age 54.5 (SD 5.9)	1. Intervention:	1. Months 0, 2 and 4 visits	2. Sexual health (sexual summary subscale of CARES)
		• > 1 menopausal symptom as moderate or severe	Individualized plans of education, counseling, pharmacologic and/or behavioral interventions, psychosocial support, referrals	2. Months 0 and 4 visits	
			2. Usual care		
Germino (2013) [56]	To test an uncertainty management intervention in young survivors	• Sample size = 313	Two groups:	10-month intervention:	1. Sexual function (Medical Outcomes Study – Sexual Functioning)
		• Mean age 44	1. Uncertainty management strategies: CD on cognitive and behavioral strategies, written guide booklet on long-term treatment effects, breast cancer resource guide, calls by nurse	1. Weekly 20-min calls x 4	
				2. Weekly 20-min calls x 4	
			2. Attention control: Calls by psychology graduate students to talk about cancer experience but no advice offered		
Greer (1992) [59]	To test psychological therapy on quality of life	• Sample size = 174	Two groups:	8-week intervention, 4-month follow up:	1. Sexual relationships (subscale of Psychological Adjustment to Illness Scale)
		• Mean age 51 (SD 13.6) in therapy group; 52 (SD 11.7) in the control group	1. Psychological therapy: Cognitive behavioral therapy for coping with cancer	1. Weekly sessions x 8	
		• All cancers except central nervous system and non-melanoma skin cancer (52 % breast cancer)	2. Controls: No therapy		
		• Psychological morbidity			
Jun (2011) [49]	To test a sexual life reframing program on marital intimacy, body image, and sexual function	• Sample size = 60	Two groups:	6-week intervention:	1. Marital intimacy (Martial Intimacy Questionnaire)
		• Mean age 45.7 (SD 6.4) in intervention group; 46.2 (SD 6.9) in control group	1. Sexual reframing program: Group sessions of up to 10 women; Sessions on relaxation, perception of problem, exposure, solving problems, acceptance, reframing	1. Weekly 2 h sessions x 6	
					2. Sexual interest (subscale, CARES)
					3. Sexual dysfunction (subscale, CARES)
		• Married with male partner			
					4. Sexual satisfaction (Sexual Satisfaction Scale)
			2. Usual care: Offered intervention for 2 h after final data collection		
Marcus (2010) [57]	To test a telephone counseling program on psychosocial outcomes	• Sample size = 304	Two groups:	12-month intervention, 18-month follow up:	5. Sexual function, (behavioral, evaluative and body image subscales of Sexual Dysfunction Scale)
		• Age < 50: 49 %	1. Telephone Counseling: Booklet with community breast cancer resources; telephone sessions with counselors; Wellness Kit with 6 thematic booklets, 2 progressive relaxation tapes, stress management guide; cognition- and emotion-focused worksheets		
		• Recent primary cancer treatment completion		1. 45-min telephone sessions: biweekly x 10, then monthly x 6	
			2. Control: Booklet with community breast cancer resources		
Rowland (2009) [51]	To test a psycho-educational group intervention on sexuality and intimacy	• Sample size = 411	Two groups:	6-week intervention:	Likert scales:
		• Mean age 57, range 35-86	1. Intervention: Group therapy led by social workers on education, communication training, sensate sex therapy	1. Weekly group sessions x 6	1. Satisfaction with variety of sexual activities
		• Distress with sexuality or intimacy, body image, and/or communication with partner			
					2. Relationship satisfaction
					3. Dyspareunia
			2. Control: Educational pamphlet on cancer survivorship		4. Pain interferes with pleasure
					5. Improved comfort with sexuality
Salonen (2009) [58]	To test a telephone-based social support intervention on quality of life	• Sample size = 250	Two groups:	1-time intervention, 2-week follow-up:	1. Sexual functioning (subscale, EORTC QLQ-BR23)
		• Mean age 56-57, range 24-75	1. Telephone support by physiotherapist: education about illness, at-home exercises, counseling on stress-related problems, exploring patient demands and exercises	1. 1-week after breast surgery phone call with therapist (length 3-25 min)	
		• Newly diagnosed with breast cancer			
			2. Control: No telephone support		
Schover (2006) [52]	To test peer counseling on improving sexual function, knowledge about reproductive health, menopausal symptoms and infertility-related distress	• Sample size = 60	Two groups:	Immediate counseling intervention, 3-month follow up:	1. Sexual Dysfunction (FSFI)
		• Mean age 49.2, range 30-77	1. Intervention: In-person peer counselor sessions reviewing Sisters Peer Intervention in Reproductive Issues after Treatment (SPIRIT)		
				1. 60-90 min peer counseling sessions x 3	
		• African American			
			2. Control: Waitlist control with SPIRIT and peer counseling at the end of study		
Schover (2011) [53]	To test peer counseling on improving sexual function, knowledge about reproductive health, menopausal symptoms and infertility-related distress	• Sample size = 300	Two groups:	6-week intervention, 6-month, 12-month follow up:	1. Sexual Dysfunction (FSFI)
		• Mean age 54.4 (SD 9.7) for peer group; 54.0 (SD 9.8) for telephone group	1. Intervention: In-person peer counselor sessions reviewing Sisters Peer Intervention in Reproductive Issues after Treatment (SPIRIT)		
				1. 60-90 min peer counseling sessions x 3	
		• African American			
				2. 30 min call to counselor encouraged x 1	
			2. Control: Telephone counseling and SPIRIT workbook		
Vos (2004) [60]	To test a group intervention (group psycho-therapy or social support) on psychosocial adjustment	• Sample size = 87	Three groups:	12-week intervention, 3-month follow up:	1. Sexual function (subscale, EORTC QLQ-BR32)
		• Mean age 49.2, range 29-68	1. Psychotherapy: Group therapy with cognitive behavior components	1. Weekly 2.5 h sessions x 12; post-treatment 1 and 2 month 2.5 h sessions	
		• Newly diagnosed with breast cancer			
			2. Social support: Group therapy with peer support		
				2. Weekly 2.5 h sessions x 12; post-treatment 1 and 2 month 2.5 h sessions	
			3. Waitlist control		
			1 & 2 Group interventions discussed fear of recurrence, coping, body image, sexuality, intimacy, social support.		
Couples-based Counseling and Educational Interventions					
Baucom (2009) [47]	To test couple-based relationship enhancement on relational distress	• Sample size = 14	Two groups:	12-week intervention, 12-month follow up:	1. Marriage quality (Quality of Marriage Index)
		• Median age 50, range 30-80	1. Relationship enhancement intervention: Cognitive behavioral therapy on cancer-related topics		
		• Married with male partner		1. Biweekly 75 min sessions with therapist x 6	2. Sexual function (Derogatis Inventory of Sexual Functioning)
			2. Controls: Community resources list		
Christensen (1983) [61]	To test a structured couples treatment program on psychosocial discomfort	• Sample size = 20	Two groups:	6-week intervention:	1. Sexual satisfaction (Sexual Satisfaction Scale)
		• Mean age 39.7	1. Therapy sessions on communication and problem solving	1. Weekly sessions x 4	
		• Married with male partner			
		• Recent mastectomy	2. Controls: No therapy		
Kalaitzi (2007) [50]	To test combined couples and sexual therapies on sexual and body image problems	• Sample size = 40	Two groups:	12-week intervention:	1. Sexual desire frequency
		• Mean age 51.8 for intervention group, 53.3 for control group	1. Intervention: Therapy sessions - 1^st^ in hospital; communication training, sensate focus, body imagery, therapist separation	1. Biweekly sessions x 6	2. Intercourse frequency
					3. Masturbation frequency
		• Married and sexually active with male partner			4. Orgasm frequency
					5. Initiative for sex
		• Recent simple mastectomy	2. Control: no therapy		6. Satisfaction with relationship
Schover (2013) [54]	To test an Internet-based intervention, with and without sexual counseling, on sexual function and satisfaction	• Sample size = 72	Two groups:	12-week intervention, 6-month follow up:	1. Sexual function (FSFI)
		• Mean age 53 (SD 9)	1. Intervention: In-person counseling to review website and behavioral homework (both partners)	1. Counseling sessions x 3	2. Sexual satisfaction (Menopausal Sexual Interest Questionnaire)
		• History of breast (80 %) or gynecologic cancer			
		• Sexually active	2. Self-help controls		
		• Sexual dysfunction (FSFI score < 26.5)	Both groups: Website on sexual and fertility consequences of cancer, genital anatomy, management of vaginal dryness, communication, dating, treatments for loss of desire, resuming sex comfortably.		
		• In a partnered relationship			
Scott (2004) [96]	To test a couples-based intervention on adjustment to cancer	• Sample size = 94	Three groups:	6-month intervention, 12-month follow up:	1. Sexual self schema (Sexual Self Schema Scale)
		• Mean age 51 (SD 9.8)	1. Couple coping training: Booklet, in-person couples counseling on coping and support		
		• Newly diagnosed breast (61 %) or gynecologic cancer		1. 2-h counseling sessions at baseline, 1-week, 5-weeks, 6 months; telephone calls at 1 and 3 months	2. Sexual desire (subscale, Brief Index of Sexual Function)
		• In a partnered relationship	2. Medical information education: Booklet on cancer and brief telephone calls		3. Sexual arousal (subscale, Brief Index of Sexual Function)
				2. Telephone calls (<15 min) at baseline, 1- and 2-week post-surgery, 6 and 9 months	
			3. Patient coping training: Booklet and in-person counseling, telephone calls on coping and support		
				3. 2-h counseling sessions at baseline, post-surgery, 1-week, 6 months; telephone calls at 1 and 3 months	

^a^All studies were randomized clinical trials of women with breast cancer (unless otherwise noted)

**Table 6:** Summary of findings

Systemic Therapy Interventions						
Reference	Outcomes	Intervention results	Control results	Comparisons	Quality of evidence (GRADE)	Comments
Barton (2007) [34]	All measures normalized to 100 point scale:	Mean change (95 % CI):	Mean change (95 % CI):	1. *p* = 0.58	Moderate	• Dropout: 12 %
		1. 5.5 (2.2-8.8)	1. 4.4 (2.4-6.5)	2. *p* = 0.11		• Side effects and quality of life did not differ by group.
	1. Sexual desire (CSFQ desire subscale)	2. 9.4 (7.0-11.2)	2. 4.7 (0.4-9.0)	3. *p* = 0.14		
	2. Pleasure (CSFQ pleasure subscale)	3. 5.7 (4.1-10.6)	3. 3.4 (2.1-6.8)	4. *p* < 0.001 for both measures		
	3. Sexual function (CSFQ total score)	4. Total 92.8 (74.9-110.7)	4. Total 1.2 (-1.8-4.3)	5. *p* = 0.82		
	4. Serum testosterone, ng/dL	Free 1.6	Free 0.18 (-0.1-0.5)	6. *p* = 0.11		
	5. Serum estradiol, pg/mL	(1.2-2.0)	5. 0.5 (-5.2-6.1)	7. *p* = 0.93		
	6. Serum SHBG, nmol/L	5. -0.3 (-2.9-2.4)	6. -0.3 (-3.0-2.3)			
	7. Serum AST, U/L	6. -3.1 (-5.1- -1.0)	7. 0.2 (-1.1-1.5)			
		7. -0.23 (-1.3-0.8)				
Buijs (2009) [42]	1. Sexual function (Sexual Activity Questionnaire)	1. Venlafaxine: No change	1. No control	1. Not reported	Moderate	• Dropout: 33 %
		Clonidine: No change				
Nunez (2013) [41]	1. Sexual function (Arizona Sexual Experience Scale)	Pre- to post- difference (SD):	Pre- to post- difference (SD):	1. *p* = 0.5	Moderate	• Dropout: 11 %
		1. 1.4 (3.8)	1. 0.6 (3.4)			
*Physical activity interventions*						
Berglund (1994) [45]	1. Sexual problems frequency (Scale 0-4)	Pre, post, 3-month mean scores (SD):	Pre, post, 3-month mean scores (SD):	1. Not significant	High	• Dropout: 8 %
		1. 0.7 (1.2), 0.6 (1.0), 0.5 (1.0)	1. 0.6 (1.0), 0.5 (0.9), 0.4 (0.7)			
Duijts (2012) [43]	1. Sexual function (Sexual Activity Questionnaire, habit subscale)	Baseline, 12-week mean scores (SD):	Baseline, 12-week mean scores (SD):	Effect size (intervention to waitlist control at 12 weeks):	Moderate	• Dropout: 17 %
						• Significant under-compliance:
		1. CBT: 0.3 (0.8), 0.5 (0.8)	1. 0.6 (0.8), 0.6 (0.8)	1. CBT: 0.31, *p* = 0.13		
		PE: 0.6 (0.8), 0.6 (0.8)		PE: 0.01,		58 % CBT; 64 % PE;
		CBT/PE: 0.4 (0.8), 0.5 (0.8)		*p* = 0.97		70 % CBT/PE
				CBT/PE: 0.15,		
				*p* = 0.44		
Speck (2010) [44]	1. Self-perception of appearance and sexuality (Body Image Relationships Scale appearance and sexuality subscale)	Mean % change (SD):	Mean % change (SD):	1. *p* = 0.004	Moderate	• Dropout: 21 %
		1. 7.3 (16.6)	1. -0.7 (18.1)			
Individual-based counseling and educational interventions						
Allen (2002) [55]	1. Sexual health (CARES sexual subscale)	Baseline, 4-month mean scores (SD):	Baseline, 4-month mean scores (SD):	1. *p* > 0.05	High	• Dropout: 9 %
				2. *p* > 0.05		
	2. Marital relationship (CARES marital subscale)	1. 2.2 (1.1), 2.1 (0.9)	1. 2.0 (1.0), 2.0 (0.9)			
		2. 1.8 (0.8), 1.7 (0.7)	2. 1.6 (0.7), 1.5 (0.6)			
Anderson (2015) [46]	1. Sexual function (sexual function subscale, Greene Climacteric Scale)	Baseline, 12-week mean score (SD) and effect size Cohen’s *d*:	Baseline, 12-week mean score (SD) and effect size Cohen’s *d*:	1. *p* = 0.05;	Moderate	• Dropout: 9 %
				Cohen’s *d*_*2*_ of post-intervention scores = 0.10		
		1. 2.0 (1.0), 1.3 (1.0)	3. 1.6 (1.1), 1.4 (1.0)			
		*d* = 0.65	*d* = 0.18			
Ganz (2000) [48]	1. Menopausal Symptom Scale Score (hot flash, vaginal and urinary subscales of BCPT Symptom Checklist)	Mean change score (95 % CI):	Mean change	1. *p* < 0.01	Moderate	• Dropout: 5 %
		1. 0.57	score (95 % CI):	2. *p* = 0.03		• Both groups used educational materials.
		(0.40-0.74)	1. 0.09			
	2. Sexual health (sexual summary subscale of CARES)	2. 0.46	(−0.04-0.21)			• Intervention group more likely to receive pharmaco-logic and behavioral interventions.
		(0.30-0.62)	2. 0.11			
			(−0.16-0.38)			
Germino (2013) [56]	1. Sexual function (Medical Outcomes Study – Sexual Functioning)	Mean score (SD) at baseline, 4-6 months, 8-10 months:	Mean score (SD) at baseline, 4-6 months, 8-10 months:	1. *p* = 0.03 at 4-6 months follow up	Moderate	• Dropout: None
						• Single time point statistically different without adjustment for baseline differences.
		1. 2.1 (1.0), 2.0 (1.0), 2.0 (1.1)	1. 2.3 (1.1), 2.3 (1.1), 2.2 (1.1)			
Greer (1992)	1. Sexual relationships (subscale of Psychological Adjustment to Illness Scale)	Mean difference (SD) from baseline to 8-weeks, to 4-months:	Mean difference (SD) from baseline to 8-weeks, to 4- months:	1. *p* = 0.53 at 8-week, *p* = 0.47 at 4-months	Moderate	• Dropout: 21 %
		1. 0.7 (7.2), -1.3 (7.7)	1. -0.4 (8.1), -1.4 (8.2)			
Jun (2011) [49]	1. Marital intimacy (Martial Intimacy Questionnaire)	Change in mean (SD) scores:	Change in mean (SD) scores:	1. *p* = 0.29	Low	• Dropout: 25 %
		1. +2.0 (5.0)	1. +0.6 (2.1)	2. *p* = 0.45		
	2. Sexual interest (subscale, CARES)	2. -0.2 (0.6)	2. -0.1 (0.9)	3. *p* = 0.53		
	3. Sexual dysfunction (subscale, CARES)	3. -0.1 (1.0)	3. +0.1 (1.1)	4. *p* < 0.001		
	4. Sexual satisfaction (Sexual Satisfaction Scale)	4. +5.3 (9.0)	4. -3.4 (5.8)			
Marcus (2010) [57]	1. Sexual function, (behavioral, evaluative and body image subscales of Sexual Dysfunction Scale)	Baseline, 12- and 18-month mean scores (approximated from figure):	Baseline, 12- and 18-month mean scores (approximated from graph):	Comparison by intervention group:	Moderate	• Dropout: 20 %
		1. 47, 40, 40	1. 45, 43, 43	1. *p* = 0.03 at 12-month,		
				*p* = 0.04 at 18-month		
Rowland (2009) [51]	Likert scales:	Mean change in score (SD):	Mean change in score (SD):	Per-protocol comparisons:	Low to very low	• Dropout: 56 %
	1. Satisfaction with variety of sexual activities	1. 0.1 (1.2)	1. -0.03 (1.0)	1. *p* = 0.23		• Intervention group: 89/284 (29 %) agreed to participate; 72/284 (25 %) attended ≥ 1 session.
		2. 0 (1.5)	2. -0.3 (1.0)	2. *p* = 0.02		
	2. Relationship satisfaction	3. 0.7 (1.5)	3. -0.1 (1.7)	3. *p* = 0.09		
	3. Dyspareunia	4. 0.3 (1.4)	4. 0 (1.1)	4. *p* = 0.29		
	4. Pain interferes with pleasure	5. Not reported	5. Not reported	5. *p* = 0.03		
	5. Improved comfort with sexuality					
Salonen (2009) [58]	1. Sexual functioning (subscale 0-100, EORTC QLQ-BR23)	Mean score (SD):	Mean score (SD):	1. *p* = 0.2	High	• Dropout: 9 %
		1. 29 (26)	1. 24 (22)			
Schover (2006) [52]	1. Sexual Dysfunction (FSFI)	1. Not reported	1. Not reported	1. No difference	Very low	• Dropout: 20 %
Schover (2011) [53]	1. Sexual Dysfunction (FSFI)	1. Not reported	1. Not reported	1. No difference	Very low	• Dropout: 38 %
Vos (2004) [60]	1. Sexual function (subscale, QLQ-BR32)	1. Not reported	1. Not reported	1. Regression coefficient comparing intervention to control: -0.17 (*p* > 0.05)	Low	• Dropout: 21 %
Couple-based counseling and educational interventions						
Baucom (2009) [47]	1. Marriage quality (Quality of Marriage Index)	Baseline, 12-week, 12-month mean score (SD):	Baseline, 12-week, 12-month mean score (SD):	Effect size of treatment to controls:	Low	• Dropout: 14 %
	2. Sexual function (Derogatis Inventory of Sexual Functioning)	1. Female: 34.0 (13.6), 39.3 (4.7), 39.7 (3.5)	1. Female: 40.8 (6.0), 42.2 (4.1), 40.2 (5.1)	Baseline to 12-weeks		
		Male: 39.3 (6.6), 39.6 (5.9), 39.6 (5.2)	Male: 42.5 (3.0), 37.5 (13.6), 41.0 (6.2)	1. Female 0.48		
				Male 0.64		
		2. Female: 11.5 (5.0), 12.7 (4.1), 13.0 (3.1)	2. Female: 10.3 (4.8), 9.8 (5.8), 9.8 (5.9)	2. Female 0.34		
				Male 0.38		
		Male: 12.4 (1.5), 13.9 (2.8), 13.2 (2.4)	Male: 12.0 (3.9), 12.3 (2.8), 9.4 (4.5)	Baseline to 12-months		
				1. Female 0.77		
				Male 0.34		
				2. Female 0.42		
				Male 1.04		
Christensen (1983) [61]	1. Sexual satisfaction (Sexual Satisfaction Scale)	Post-test mean score (SD):	Post-test mean score (SD):	1. *p* < 0.05 for both partners	High	• Dropout: None
		1. Female partner: 80.4 (31.5)	1. Female partner: 69.0 (20.2)			
		Male partner: 81.3 (28.7)	Male partner: 67.3 (28.6)			
Kalaitzi (2007) [50]	1. Sexual desire frequency	Baseline, 12-week mean scores (95 % CI):	Baseline, 12-week mean scores (95 % CI):	1. *p* = 0.73		• Dropout: None
	2. Intercourse frequency			2. *p* = 0.14		
	3. Masturbation frequency	1. 2.9 (2.3-3.4), 2.8 (2.3-3.2)	1. 3.0 (2.6-3.4), 2.7 (2.2-3.1)	3. *p* = 0.32		
	4. Orgasm frequency	2. 3.2 (2.7-3.6), 2.9 (2.5-3.3)	2. 3.2 (3.0-3.4), 2.5 (2.1-2.9)	4. *p* = 0.03		
	5. Initiative for sex	3. 1.9 (1.4-2.3), 1.6 (1.2-2.0)	3. 1.9 (1.6-2.2), 1.9 (1.5-2.2)	5. *p* < 0.001		
	6. Satisfaction with relationship	4. 3.3 (2.8-3.8), 3.7 (3.4-4.0)	4. 3.6 (3.1-4.0), 3.1 (2.6-3.6)	6. *p* = 0.01		
		5. 2.2 (1.8-2.6), 2.7 (2.3-3.0)	5. 2.6 (2.2-3.0), 1.8 (1.4-2.2)			
		6. 3.8 (3.3-4.2), 4.5 (4.2-4.7)	6. 3.3 (2.9-3.7), 3.7 (3.2-4.1)			
Schover (2013) [54]	1. Sexual function (FSFI)	Linear mixed model coefficients, post-treatment versus baseline:	Linear mixed model coefficients, post-treatment versus baseline:	1. *p* = 0.024	Low	• Dropout: 36 %
	2. Sexual satisfaction (Menopausal Sexual Interest Questionnaire)			2. *p* = 0.01		
		1. 7.4	1. 2.8			
		2. 13.2	2. 3.4			
Scott (2004) [96]	1. Sexual Self Schema Scale	Baseline, post-treatment mean score(SD):	Baseline, post-treatment mean score (SD):	Effect size, p-value of couples coping vs other two conditions:	Moderate	• Dropout: 11 %
	2. Sexual desire (subscale, Brief Index of Sexual Function)	Couples coping	Medical information	1. d = 0.8, *p* < 0.05		
	3. Sexual arousal (subscale, Brief Index of Sexual Function)	1. 57.3 (13.5), 62.8 (12.2)	1. 55.4 (14.3), 55.8 (11.0)	2. No difference		
		2. 4.2 (2.8), 4.0 (2.3)	2. 3.1 (1.9), 2.4 (2.4)	3. No difference		
		3. 2.7 (2.5), 2.0 (2.0)	3. 1.9 (1.4), 1.6 (1.6)			
			Patient coping			
			1. 55.7 (14.3), 56.0 (12.0)			
			2. 3.7 (2.6), 2.7 (2.9)			
			3. 3.1 (1.6), 2.2 (2.3)			

### Physical therapy interventions

Three RCTs tested physical activity interventions on the primary outcomes of hot flashes, lymphedema, or physical strength and measured sexual health secondarily (Tables 5 and 6, Fig 2c). All participants completed primary breast cancer treatment. There were no studies on pelvic floor physical therapy. Included studies were conducted in the Netherlands, Sweden and United States. A home-based, self-directed exercise program intervened on 422 BCS and did not improve sexual habit, frequency or discomfort as measured by the Sexual Activity Questionnaire [43]. In the two arms with cognitive behavioral therapy, with or without exercise, there was a modest effect on improving sexual health habit at 24 weeks when compared to waitlist controls. Strength training over one year in the second trial of 295 participants was associated with a small improvement in self-perceptions of appearance and sexuality [44]. Finally, a general physical training and coping skills intervention in 199 cancer survivors (80 % with breast cancer) did not directly address sexual health and did not find change in frequency of sexual problems [45].

### Counseling and educational interventions

Seventeen RCTs delivered counseling and/or educational interventions and measured sexual health outcomes in a total of 2,494 participants (Tables 5 and 6, Fig 2d). Participants were studied at various stages of cancer treatment. Studies were conducted in Australia, Finland, Greece, Korea, Netherlands, United Kingdom, and United States. Nine studies targeted sexual health as the primary outcome [46–54]. There was considerable heterogeneity on intervention and outcome measurements. Twelve studies intervened on the individual, while 5 studies intervened on the couple. The majority delivered in-person interventions, many with additional telephone-support [46, 53, 55–58]. Two recent studies tested web-based interventions [46, 54]. Counseling strategies varied widely, from problem-solving therapy to sexual therapy to cognitive behavioral therapy. Most interventions were delivered by nurses, psychologists, social workers, or peers.

Several findings were consistent. In studies designed specifically to intervene on sexual health, improvements in sexual function were observed in the intervention group compared to controls [46, 48–51], but effect sizes were generally modest and of unclear clinical significance. For example, a 4-month trial tested behavioral and non-estrogen replacement pharmacologic interventions on menopausal symptoms in 76 BCS [48]. The intervention group received individualized plans of education, counseling, pharmacologic and/or behavioral interventions, psychosocial support, and referrals compared to controls who underwent usual care. Sexual function was measured by the CARES Sexual Summary Scale, which is scored from 0 to 4 (higher score indicating more severe problems). The mean score change of the intervention group (0.46, 95 % CI 0.30–0.62) was statistically significantly larger than that of the control group (0.11, 95 % CI −0.16 to 0.38), *p* = 0.03, but clinical relevance is unclear. Most studies intervening on general psychosocial health, rather than targeting sexual health, did not appear to improve sexual function [55, 58–60]. Researchers who undertook group therapy interventions reported difficulties with attendance and higher dropout rates [49, 51]. Couple-based therapy incorporated counseling on cancer, sexual health, and communication and consistently improved various aspects of sexual function, most frequently sexual satisfaction [47, 50, 61].

## Discussion

The majority of BCS experience sexual problems in survivorship, most commonly vaginal and vulvar dryness. Despite the significant population of BCS and high prevalence of sexual problems, the number of RCTs intervening on sexual health was limited. This review summarized evidence for BCS across all ages, because trials in midlife BCS were few. Results showed significant evidence for regular use of vaginal moisturizers to improve dryness, dyspareunia, and sexual satisfaction. Uncontrolled studies with vaginal estradiol, estriol or testosterone also improved vaginal symptoms, but showed systemic absorption. Educational and counseling interventions, particularly those targeting sexual dysfunction, improved various aspects of sexual health. No consistent improvements in sexual health were observed with physical activity, transdermal testosterone or hot flash interventions. Overall for most included studies, the quality of evidence by GRADE criteria was moderate to low.

Vulvovaginal symptoms occur in 20 to 50 % of healthy women of midlife and older as a result of estrogen deprivation [62]. BCS are at heightened risk of these symptoms because chemotherapy, oophorectomy and/or endocrine therapies further decrease estrogen exposure. The clinical trial data show improvements in vaginal dryness, dyspareunia, sexual satisfaction and frequency, and vaginal pH with regular use at least 2-3 times weekly of a polycarbophil-based vaginal moisturizer. Compliance for at least twelve weeks is important, because major symptom gains occurred between 1-3 months and recur after stopping use, similar to data in the general population [63]. Vulvovaginal symptom relief from regular use of other moisturizers is likely, and pH balance in products may be important [23, 29]. Among available vaginal moisturizers, BCS should consider preferentially using products with evidence of efficacy.

Use of minimally absorbed local vaginal estrogens and androgens provide vaginal symptom relief, with local estrogens appearing more effective than non-hormonal moisturizers [24, 64]. Even at low doses, estradiol tablets and creams and compounded testosterone are systemically absorbed [24, 28, 30–33]. Unfortunately, there are no clinical trial data on adverse breast cancer outcomes with extended use. Nor are there studies in BCS that compare 7, 10 and 25 micrograms of vaginal estradiol for symptom control and systemic absorption. Whether risk of breast cancer recurrence or death would be higher in estrogen-responsive tumors is also unknown. As local estrogens and androgens are not FDA-approved for use in BCS, these medications are prescribed off-label and use requires careful discussion between BCS and their healthcare providers.

There was a lack of evidence to support incorporating systemic interventions or physical therapy into the treatment paradigm for sexual dysfunction. The single trial on transdermal testosterone did not demonstrate greater sexual desire compared to the placebo cream after 1 month of use [34]. These findings stand in contrast to several trials in women without prior breast cancer in which androgen therapy improved sexual desire, potentially because these trials were longer in duration (12-24 weeks) and provided supplemental estrogen [35–40]. Notably, there were no clinical trials on treating sexual dysfunction related to serotonin receptor uptake inhibitors in BCS.

Multiple counseling and educational strategies, particularly those targeting sexual dysfunction, have been shown to improve sexual health in BCS. Marriage and family therapists, sex therapists, sexual counselors or psychologists offer counseling interventions. With the aid of online resources, BCS can look for providers who are appropriately educated, credentialed or have significant prior experience with sexual health after cancer. Excellent online resources are found on sites for the American Association of Sexuality Educators, Counselors, and Therapists, the International Society for the Study of Women’s Sexual Health, and the American Cancer Society. A number of investigators have designed educational interventions using printed materials, CDs, and websites for content with healthcare provider or peer support [46, 48, 52–54]. This approach is important to study further, as it has the potential advantage of being delivered remotely to extend access to BCS who do not have specialized care locally.

The strength of this review is the systematic approach to identifying and grading current evidence on sexual health interventions specific to breast cancer survivors. This approach enabled us to identify the gaps in data. Several interventions that have shown promise in women without a history of breast cancer have not undergone clinical trials in BCS. These include ospemifene and systemic DHEA for the treatment of vulvovaginal symptoms and flibanserin for the treatment of arousal and sexual interest disorders [65, 66]. The primary limitation was heterogeneity of interventions and outcome measures that restricted the ability to pool data from studies of limited sample size. A recent systematic review sought to evaluate the psychometric properties of sexual dysfunction screening tools and the extent to which they measure DSM-5 aspects of sexual dysfunction for BCS [67]. The review found 31 different scales measuring sexual function, of which the Arizona Sexual Experience Scale, Female Sexual Function Index, and Sexual Problems Scale were determined to meet criteria for acceptable psychometric properties while incorporating DSM-5 areas of sexual dysfunction. Future studies in BCS should carefully consider these outcome measures in study design.

This review demonstrated that current evidence on interventions for improving sexual interest, orgasm and genitopelvic pain in BCS of midlife is limited in quantity and moderate to low in quality. From these data, we recommend prolonged and regular use of non-hormonal vaginal moisturizers to alleviate vulvar and vaginal dryness symptoms and dyspareunia. We also recommend seeking educational and counseling interventions. A number of online resources on sexual health after breast cancer may be useful for BCS and their providers (Table 7). Because each of these interventions have limited efficacy, clinical trials to test novel interventions such as ospemifene are needed in breast cancer survivors.

**Table 7:** Patient Resources: Companion document for use by women seeking management for female sexual dysfunction

What type of information is here?	What organization provides this resource?	Link to website
Fact sheet and video describing sexual problems and treatment options for the general population.	American Society for Reproductive Medicine	http://www.reproductivefacts.org/FACTSHEET_Sexual_Dysfunction_and_Infertility/
		http://www.reproductivefacts.org/awards/detail.aspx?id=10701
Fact sheets about cancer, its effect on sex and sexuality, and treatment options	American Cancer Society	http://www.cancer.org/treatment/treatmentsandsideeffects/physicalsideeffects/sexualsideeffectsinwomen/sexualityforthewoman/index
Sexual function screening guidelines and treatment options for cancer survivors	National Comprehensive Cancer Network	http://www.nccn.org/professionals/physician_gls/pdf/survivorship.pdf
Recorded talk by sexual health providers on rediscovering intimacy after cancer treatment	Cancer Care, in collaboration with National Cancer Institute, Livestrong, LBBC, Intercultural Cancer Council, National Coalition for Cancer Survivorship	http://www.cancercare.org/connect_workshops/138-cancer_survivorship_2008-05-13
Information on vaginal dryness treatment options for the general population	North American Menopause Society	http://www.menopause.org/docs/for-women/mndryness.pdf
Online forum to talk to other breast cancer survivors about sexual concerns	BreastCancer.org	http://www.breastcancer.org/tips/intimacy
Finding a sexual health provider	American Association of Sexuality Educations, Counselors, and Therapists	http://www.aasect.org/referral-directory
Finding a sexual health provider	Society for Sex Therapy and Research	http://www.sstarnet.org/therapist-directory.php

## Appendix

**PubMed Search Strategy**

(("Clinical Trial"[Publication Type] OR "Comparative Study"[Publication Type]) OR ("Phase I Clinical Trial" OR "Phase II Clinical Trial" OR "Phase III Clinical Trial" OR "Phase IV Clinical Trial" OR "Controlled Clinical Trial" OR "Multicenter Study" OR "Observational Study" OR "Randomized Controlled Trial" OR "Pragmatic Clinical Trial" OR "Comparative Study")).

AND (("Breast Neoplasms"[Mesh] NOT "Breast Neoplasms, Male"[Mesh]) OR ("Breast cancer" OR "Breast Neoplasms")).

AND (("Sexual Dysfunctions, Psychological/prevention and control"[Mesh] OR "Sexual Dysfunctions, Psychological/rehabilitation"[Mesh] OR "Sexual Dysfunctions, Psychological/therapy"[Mesh] OR "Sexual Dysfunction, Physiological/prevention and control"[Mesh] OR "Sexual Dysfunction, Physiological/rehabilitation"[Mesh] OR "Sexual Dysfunction, Physiological/therapy"[Mesh] OR "Vaginal Creams, Foams, and Jellies"[Mesh] OR "Biofeedback, Psychology"[Mesh] OR "Cognitive Therapy"[Mesh] OR "Psychotherapy"[Mesh] OR "Sex Counseling"[Mesh] OR "Patient Education as Topic"[Mesh] OR "Testosterone/therapy"[Mesh] OR "Antidepressive Agents"[Mesh]) OR ("vaginal lubricant" OR "vaginal moisturizer" OR "pelvic floor muscle relaxation" OR "pelvic floor physical therapy" OR "pelvic floor muscle training" OR "biofeedback" OR "vaginal dilator" OR "biofeedback" OR "cognitive behavioral therapy" OR ("sex" AND "therapy") OR "psychotherapy" OR "sex counseling" OR "patient education" OR "testosterone" OR "antidepressant" OR "treatment" OR "flibanserin" OR "ospemifene" OR "vibrator" OR "vaginal dilator" OR "DHEA")).

AND (("Sexual Dysfunction, Physiological"[Mesh] OR "Interpersonal Relations"[Mesh] OR "Sexual Behavior"[Mesh] OR "Vaginismus"[Mesh] OR "Coitus"[Mesh] OR "Libido"[Mesh] OR "Orgasm"[Mesh]) OR ("dyspareunia" OR "coitus" OR "coital frequency" OR "intercourse frequency" OR "vaginal dryness" OR "sexual lubrication" OR "vulvovaginal atrophy" OR "libido" OR "sexual function" OR "sexual dysfunction" OR "sexual interest" OR "sexual desire" OR "sexual arousal" OR "orgasm" OR "sexual pleasure" OR "sexual dissatisfaction" OR "sex" OR "intimacy" OR "sexual desire disorder" OR "sexual arousal disorder" OR "orgasmic disorder" OR "sexual pain disorder")).
